# An Uneven Playing Field: Demographic and Regionalized Disparities in Access to Device-Based Therapies for Cardiogenic Shock

**DOI:** 10.1016/j.jscai.2023.101220

**Published:** 2023-11-10

**Authors:** Behnam N. Tehrani, Kelly C. Epps, Wayne B. Batchelor

**Affiliations:** Inova Schar Heart and Vascular Institute, Falls Church, Virginia

**Keywords:** cardiogenic shock, disparities in access, mechanical circulatory support

With advances in primary percutaneous coronary intervention and the integration of regionalized systems of care, in-hospital mortality following acute myocardial infarction (AMI) has dropped to <5%^1^ However, in the 5 to 12% of AMI complicated by circulatory collapse due to cardiogenic shock (CS), short-term mortality rates remain >40%.^2^, ^3^, ^4^ In response to these discouraging outcomes, an increasing number of patients with CS due to AMI or acutely decompensated heart failure undergo implantation of temporizing mechanical circulatory support (tMCS) devices to support hemodynamics and end-organ perfusion as a bridge to myocardial recovery or advanced cardiac replacement therapies.^5^^,^^6^ More recently, this surge in CS device-based therapy has been fueled by an increase in the availability of microaxial left ventricular assist devices (mLVAD) and extracorporeal membrane oxygenation (ECMO), both of which provide greater hemometabolic support than a conventional intraaortic balloon pump (IABP).^7^, ^8^, ^9^ This trend exists despite an absence of randomized clinical trial (RCT) data demonstrating improvement in short-term survival with tMCS devices compared with medical therapy or IABP, and with clinical guidelines assigning their routine use in AMI-CS a Class IIb (Level of Evidence: C) recommendation.^10^, ^11^, ^12^ In part, the increased use of tMCS devices has been fueled by data from dedicated North American CS registries that show that the early use of hemodynamically tailored tMCS at institutions with appropriate levels of expertise is associated with improved survival.^13^, ^14^, ^15^ To muddy the waters in this field even further, there is marked regional variation in the expertise, access to, and use of tMCS devices across the United States for CS patients who remain unresponsive to conventional treatment strategies.^8^^,^^16^, ^17^, ^18^

How tMCS use is influenced by patient demographic characteristics, such as race, ethnicity, and social determinants of health has not been well studied. In this issue of *JSCAI,* Nathan et al^19^ examined racial, ethnic, and socioeconomic disparities in access to tMCS in Medicare beneficiaries with an admission or diagnosis discharge of CS who were treated with IABP, mLVAD, or ECMO at 1829 percutaneous coronary intervention-capable acute care facilities within the 25 largest core-based statistical areas in the United States. The authors should be commended for providing unique insight into this device-related health care disparity. Using Medicare demographic characteristics to identify race and ethnicity, and individual assessments of median household income, Medicaid dual-eligibility, and distressed community index scores to categorize socioeconomic status, the authors (1) compared patient socioeconomic and hospital characteristics of institutions with and without mLVAD or ECMO programs; (2) determined the likelihood of mLVAD or ECMO use based on race, ethnicity, and socioeconomic status; and (3) described variations in ZIP code-level age-adjusted rates of mLVAD utilization.

The findings were sobering, albeit not surprising; widespread disparities in access to these cutting-edge therapies were observed based on race, ethnicity, and rurality. More than 90% of sites with mLVAD and ECMO programs were located in metropolitan areas, and these hospitals were more likely to treat patients with high median household incomes. Only 3 centers with mLVAD and 1 with ECMO capabilities were identified in rural areas. Racial and ethnic disparities were equally stark. Less than 10% of patients receiving an mLVAD self-identifying as African American. Using generalized linear mixed effects models, associations between socioeconomic status, race, ethnicity, and the likelihood of receiving an mLVAD were elucidated. African American and dual Medicaid-eligible CS patients were 37% and 20% less, likely, respectively, to receive advanced device-based therapies. More pronounced findings were noted with ECMO, such that for each $1000 decrement in median household income there was a 35% reduction in the likelihood of receiving ECMO. African Americans and dual Medicaid eligible patients were also significantly less likely to undergo ECMO cannulation for refractory circulatory collapse. Chloropleth maps depicting device implantation rates across core-based statistical areas of the United States provided stark graphical representation of these disparities.

There is precedent for demographic and geographic variations in access to cutting-edge technologies within interventional cardiology.^20^, ^21^, ^22^, ^23^ Using health care administrative inpatient claims data, Damluji et al^20^ reported a nearly 7-fold difference in transcatheter aortic valve replacement utilization rates in the state of Florida, with the majority of dedicated valve centers located in high population density areas, and residents of low population density areas experiencing marked increases in travel time/distance and >6-fold higher procedural mortality. Similar findings were noted in a contemporary analysis of 1567 hospital discharges following transcatheter edge-to-edge repair for severe mitral valve regurgitation from the Arizona, Colorado, Florida, Maryland, North Carolina, New Jersey, New York, and Virginia State Inpatient Databases. Significant racial disparities in access to high-volume mitral valve institutions were noted, with African Americans and Hispanics being afforded 59% and 51% lower chances of access to transcatheter mitral valve repair at high-volume centers, respectively.^21^ Hispanics were also 3 times more likely to experience in-hospital mortality post-procedure.^21^ Unlike valve therapies, tMCS devices have not been associated with improved survival, and they are often implanted under emergency circumstances, in which the rates of major bleeding complications and acute limb ischemia may be as high as 40%.^24^, ^25^, ^26^, ^27^ However, given the well-established relationship between procedural volume and outcomes in CS,^16^ another unwanted impact of low tMCS device use in nonmetropolitan hospitals is that these facilities may experience worse outcomes and higher complication rates due to a relative lack of experience. In the context of recent observational data suggesting the potential for improved outcomes when tMCS devices are implanted using standardized team-based protocols, one may argue that the demographic and geographic differences in outcomes with CS seen today are in part due to the nonuniform patterns of patient selection seen in real-world clinical practice.^13^

Notwithstanding the absence of professional societal guideline support for the broad use of tMCS devices in CS, the findings of Nathan et al highlight pervasive racial and ethnic barriers to the use of advanced medical device therapies present in the US health care system. These inequities have historically and disproportionately affected segments of our society who not only have the greatest burden of health and social risk factors but who are also underrepresented in clinical trials and whose voices are often not heard^28^, ^29^, ^30^ within the cacophony of health care discussions. In the case of tMCS, further research is needed in the form of pragmatic and adequately powered RCTs enriched with broad patient subsets and prespecified treatment protocols. Multicenter device agnostic CS registries, including the American Heart Association’s Cardiogenic Shock Registry^31^ and regionalized CS networks, should provide further insight into the utilization and outcomes associated with advanced device-based therapies for CS across race, ethnicity, sex, rurality, and socioeconomic status. However, at their core, the findings of Nathan et al are disturbingly emblematic of a much more complex and challenging sociopolitical problem that is deeply rooted in the world’s richest health care system. Therefore, to truly level the “device therapy playing field” will require not only more targeted health-related outcomes research but a national commitment to improve health care access and quality, especially for those most in need of acute lifesaving therapies.
